# Recent Advances and Therapeutic Benefits of Glucagon-Like Peptide-1 (GLP-1) Agonists in the Management of Type 2 Diabetes and Associated Metabolic Disorders

**DOI:** 10.7759/cureus.72080

**Published:** 2024-10-21

**Authors:** John O Olukorode, Dolapo A Orimoloye, Nwachukwu O Nwachukwu, Chidera N Onwuzo, Praise O Oloyede, Temiloluwa Fayemi, Oluwatobi S Odunaike, Petra S Ayobami-Ojo, Nwachi Divine, Demilade J Alo, Chukwurah U Alex

**Affiliations:** 1 Internal Medicine, Babcock University Teaching Hospital, Ilishan-Remo, NGA; 2 Internal Medicine, College of Medicine, University of Lagos, Lagos, NGA; 3 Public Health, Liverpool John Moores University, Liverpool, GBR; 4 Internal Medicine, SUNY Upstate Medical University, Syracuse, USA; 5 Internal Medicine, Benjamin S. Carson College of Health and Medical Sciences, Ilishan-Remo, NGA; 6 Internal Medicine, General Hospital Lagos Island, Lagos, NGA; 7 Internal Medicine, School of Medicine and Population Health, University of Sheffield, Sheffield, GBR; 8 Internal Medicine, Danylo Halytsky Lviv National Medical University, Lviv, UKR

**Keywords:** benefits of glp-1 agonists, diabetes, glp-1 agonist, glucagon-like peptide, liraglutide, management of type 2 diabetes, obstructive sleep apnea, tirzepatide

## Abstract

Glucagon-like peptide-1 (GLP-1) agonists have emerged as a groundbreaking class of medications for managing type 2 diabetes and associated metabolic disorders. These agents not only improve glycemic control by increasing insulin secretion and reducing glucagon levels but also promote significant weight loss, enhance cardiovascular and renal health, and offer potential neuroprotective benefits. Their multifaceted mechanisms include appetite suppression, increased energy expenditure, and direct neuroprotective effects. GLP-1 agonists have shown recent benefits in Obstructive Sleep Apnea, and the treatment of neurodegenerative diseases such as Alzheimer’s and Parkinson’s, as well as reducing the risk of stroke. This review highlights the therapeutic potential of GLP-1 agonists in diabetes management and beyond, advocating for continued research to optimize their clinical use and explore new therapeutic avenues.

## Introduction and background

Glucagon-like peptide-1 (GLP-1) is a hormone secreted by intestinal L-cells in the distal ileum and colon. It stimulates insulin release in response to glucose intake by activating GLP-1 receptors on pancreatic beta cells [1]. GLP-1 receptor agonists, also known as incretin mimetics, are antidiabetic drugs that mimic GLP-1 to enhance insulin secretion, improve glycemic control, promote weight loss, and provide cardiovascular and renal benefits [2, 3].

These agonists work by increasing cyclic adenosine monophosphate (cAMP) levels within beta cells, activating protein kinase A (PKA), and initiating several mechanisms that enhance insulin secretion [1]. GLP-1 also regulates other hormones by stimulating somatostatin secretion and inhibiting glucagon secretion, which normally raises blood glucose levels [4].

The development of GLP-1 agonists began in the 1970s with the discovery of glucose-dependent insulinotropic polypeptide (GIP) and continued with the identification of GLP-1 in the early 1980s [5]. Exenatide, the first GLP-1 receptor agonist, was approved in 2005, leading to the development of other once-weekly therapies [6]. Liraglutide was approved for obesity treatment in 2014, and semaglutide, the first oral GLP-1 receptor agonist, was approved in 2019 [6, 7]. These drugs are now used to treat type 2 diabetes and following experiments on animals, are being explored for other conditions such as Obstructive Sleep Apnea (OSA), heart failure, Parkinson’s disease, and Alzheimer’s disease, etc. [7].

## Review

GLP-1 agonists in glycemic control 

GLP-1 agonists have revolutionized the management of glycemic control in patients with type 2 diabetes mellitus (T2DM). A comprehensive meta-analysis by Haiqiang Yao et al. evaluated the efficacy of 15 GLP-1 receptor agonists (GLP-1RA), including novel drugs, in lowering hemoglobin A1c (HbA1c). This study analyzed the effectiveness of glycaemic control, body weight, lipid profile, and adverse events using data from 76 eligible randomized controlled trials involving 39,246 participants. The findings indicated that all 15 GLP-1RA drugs significantly reduced HbA1c concentrations and fasting blood glucose levels compared with placebo in adults with type 2 diabetes [8]. 

Another meta-analysis by Orsini Federici et al. compared the efficacy and safety of weekly albiglutide (30-50 mg), a GLP-1 agonist, with daily sitagliptin (100 mg), a DPP-4 inhibitor, daily glimepiride (2-4 mg), a sulfonylurea, and placebo, all added to metformin in patients with T2DM. The study demonstrated that albiglutide produced superior reductions in HbA1c at 104 weeks compared with placebo, sitagliptin, or glimepiride [9].

The study further compared the efficacy and safety of weekly dulaglutide (0.75 and 1.5 mg) with daily glimepiride (1-3 mg) as monotherapy in a double-blinded randomized study involving East Asian patients with T2DM. Both doses of dulaglutide resulted in significantly greater reductions in HbA1c from baseline at 26 weeks compared to glimepiride [9].

These studies highlight the significant benefits of GLP-1 receptor agonists in improving glycemic control and lowering HbA1c levels in patients with type 2 diabetes, offering a robust comparison with other antidiabetic agents.

Weight management and obesity 

Glucagon-like peptide-1 (GLP-1) agonists were originally developed for treating type 2 diabetes, but they have also demonstrated significant weight reduction benefits, consistently observed in clinical trials and studies. The following scientific evidence substantiates this claim:

The SCALE Obesity and Prediabetes trial investigated the effects of liraglutide (a GLP-1 agonist) on weight management. This double-blind trial involved 3,731 participants with a BMI of 30 or more without type 2 diabetes or a BMI of 27 or more with treated or untreated dyslipidemia or hypertension. Participants were divided into two groups: one received lifestyle modification counseling with a placebo, and the other received 3.0mg of liraglutide along with lifestyle modification counseling. Results showed an average weight loss of 8.4 ±7.3 kg in the liraglutide group, compared to 2.8±6.5 kg in the placebo group. Additionally, 63.2% of patients in the liraglutide group lost at least 5% of their body weight, versus 27.1% in the placebo group. Moreover, 33.1% and 10.6%, respectively, lost more than 10% of their body weight [10].

The DURATION-8 trial, a randomized control trial involving 695 adults with type 2 diabetes uncontrolled with metformin, assessed the effects over 104 weeks. Participants were assigned to three groups: exenatide plus dapagliflozin group, exenatide plus placebo group, and dapagliflozin plus placebo group. At week 104, the exenatide plus dapagliflozin group showed greater reductions in HbA1C, weight, fasting plasma glucose, and 2-hour postprandial glucose, highlighting the dual benefits of exenatide (a GLP-1 agonist) in glycemic control and weight loss [11].

Wilding JPH et al. demonstrated the efficacy of Semaglutide (a GLP-1 agonist) in weight loss, with 86.4% of participants achieving a 5% reduction in body weight compared to 31.5% in the placebo group. At week 68, the mean weight loss was 15.3 kg in the semaglutide group versus 2.6 kg in the placebo group [12].

A systematic review and meta-analysis by Liu Y et al., which included 41 trials, explored the weight-loss effects of GLP-1 agonists in non-diabetic individuals with obesity or overweight. The findings provided conclusive evidence that GLP-1 agonists cause weight loss in a nonlinear dose-response manner in individuals who are overweight or obese without diabetes. They significantly reduced BMI, waist circumference, and total body fat (P < 0.0001), though no significant change in the waist-to-hip ratio was observed [13].

GLP-1 agonists induce weight loss through multiple physiological mechanisms:

Appetite Suppression: GLP-1 agonists delay gastric emptying and reduce gut motility, increasing satiety. They also bind to GLP-1 receptors on orexigenic and anorexigenic neurons, modulating central nervous system pathways involved in reward processing and motivated behaviors, leading to reduced food intake [14]. This is the main pathway through which GLP-1 agonists cause weight loss.

Increased Insulin Secretion: GLP-1 agonists enhance glucose-dependent insulin release, promoting glucose homeostasis and indirectly reducing body weight [14]. There is a net weight loss due to the loss of lean mass, as compared to fat mass [15].

Reduced Glucagon Secretion: They inhibit glucagon release, which helps lower blood glucose levels and also contributes to loss of lean mass [14, 15].

Energy Expenditure: GLP-1 agonists increase thermogenesis and improve mitochondrial function in adipose tissue, contributing to a long-term negative energy balance (lowered energy intake from reduced appetite compared to increased expenditure) [14].

Through these mechanisms, GLP-1 agonists effectively promote weight loss and are used in the management of overweight and obese individuals.

Cardiovascular benefits 

It is no news that obstructive sleep apnea may occur concomitantly with type 2 diabetes mellitus, especially in the presence of obesity; and if left untreated, has the propensity to increase the risk of cardiovascular diseases. The prevalence of Obstructive Sleep Apnea in patients with both obesity and type 2 diabetes mellitus can be as high as 86%. Thus having an oral antidiabetic that tackles both diabetes and obesity significantly reduces the risk of obstructive sleep apnea occurring and serves therapeutic effects in patients with the disease, as weight loss is one of the mainstays of management of Obstructive Sleep Apnea [16]. The Apnea-Hypopnea Index (AHI) is the total number of apneas and hypopneas that occur per hour of sleep, with mild OSA being 5 to 15 events/hour, moderate OSA - 15 to 30 events/hour and severe OSA - >30 events/hour. 

The STEP (Semaglutide Treatment Effect in People with obesity) trials showed weight loss of up to 17% of body weight with the use of semaglutide for a period of 68 weeks [17]. A randomized, double-blinded trial done by Blackman et al. in the SCALE (Satiety and Clinical Adiposity-Liraglutide Evidence in non-diabetic and diabetic individuals) sleep apnea study group showed a reduction in the severity of obstructive sleep apnea by a mean AHI of 12.2/h in non-diabetic patients treated with Liraglutide over a period of 32 weeks [18].

Tirzepatide, a newly approved combined GLP-1 and GIP agonist, achieves a mean weight reduction of 25.3% when used for 88 weeks, as shown in the SURMOUNT-4 trial, and a reduction of as high as 48-56% in the AHI when compared to placebo [19, 20].

GLP-1 agonists have cardioprotective effects in diabetic patients [21]. In non-diabetic patients, they have also been shown to reduce the risk of cardiovascular events and mortality. Semaglutide, for example, was shown by Lincoff et al. to reduce the risk of death from non-fatal myocardial infarction or stroke by 20%, due to its weight reduction properties and reduction in ectopic adipose tissue [21]. Weight loss further leads to a reduction in the inflammatory and prothrombotic events that occur due to obesity [21]. In patients with heart failure, semaglutide has been shown to cause reduced levels of N-terminal pro B-type natriuretic peptide (NT-proBNP) and C-reactive protein, thus leading to improved exercise function and quality of life [22].

Long-term use of GLP-1 agonists such as semaglutide and liraglutide has been shown to reduce blood pressure via increased natriuresis and inhibition of the renin-angiotensin-aldosterone system [23]. GLP-1 agonists have also been shown to improve vascular endothelial function with improved vascular relaxation and reduced diastolic blood pressure via the promotion of nitric oxide release and reduction in free radicals [24].

GLP-1 agonists have been shown to reduce fatty acid synthesis in the liver [25]. They have been shown to significantly increase high-density lipoprotein and reduce low-density lipoprotein and total cholesterol levels [8]. Weight loss caused by GLP-1 agonists has been shown to reduce deposits of ectopic adipose tissue which contribute to atherosclerosis [21].

Renal benefits 

The prevalence of chronic kidney disease (CKD) in individuals with type 2 diabetes (T2DM) is alarming, with 40% of patients affected. Moreover, diabetic kidney disease (DKD) is the primary driver of end-stage renal disease (ESRD), necessitating life-sustaining renal replacement therapy (RRT) [26]. 

Diabetic nephropathy (DN) is a distinctive condition characterized by intricate changes to the kidneys' structure and function in individuals with diabetes. These changes involve the thickening of the glomerular basement membrane, the proliferation of mesangial cells, and the deposition of aberrant collagen, leading to interstitial fibrosis and disruption of capillary architecture. Vascular lesions, such as hyalinosis, are also prevalent [27].

Hyperglycemia plays a pivotal role in triggering DKD, precipitating a cascade of cellular alterations that disrupt glucose metabolism, fatty acid, and amino acid ratios, mitochondrial function, and the coupling of respiratory chain proteins. Clinically, DKD manifests as nephrotic-range proteinuria, hypertension, and a gradual decline in renal function [28].

Despite a 29% decline in the incidence of type 2 diabetes (T2DM)-related end-stage renal disease (ESRD), a significant number of patients with diabetes (20 per 10,000) continue to require renal replacement therapy (RRT) annually. In fact, T2DM remains the leading cause of chronic kidney disease (CKD) and ESRD, accounting for approximately 33% of all patients initiating RRT globally [29].

The persistence of this issue shows the urgent need for innovative therapeutic approaches and evidence-based guidelines. The 2022 Kidney Disease Improving Global Outcomes (KDIGO) guidelines for patients with CKD and the 2022 American Diabetes Association (ADA) standards-of-care (SoC) guidelines for patients with T2DM recommend the use of renin-angiotensin-aldosterone system (RAAS) blocking agents and sodium-glucose co-transporter 2 inhibitors (SGLT2is) as first-line treatments for most patients [5]. Furthermore, both guidelines suggest that non-steroidal mineralocorticoid receptor antagonists (such as finerenone) may be an effective option for patients with CKD who are at high risk of cardiovascular events, CKD progression, or cannot tolerate SGLT2is [30, 31].

A case report of a 42-year-old female with type 2 diabetes (T2DM) and progressive kidney failure presents a complex therapeutic challenge. Given the proven efficacy of sodium-glucose cotransporter-2 inhibitors (SGLT2is) in reducing the risk of cardiovascular events and chronic kidney disease (CKD) progression, an SGLT2i is initially considered as a first-line treatment. However, the patient's compromised estimated glomerular filtration rate (eGFR) necessitates alternative approaches [32].

In this context, a long-acting glucagon-like peptide 1 receptor agonist (GLP1RA) is recommended as the next most effective glucose-lowering agent for patients with T2DM and CKD. The LEADER trial provides compelling evidence for the benefits of liraglutide in reducing major adverse cardiovascular events (MACE), with a strikingly greater risk reduction observed among individuals with eGFR ≥60 mL/min/1.73 m² compared to those with eGFR <60 mL/min/1.73 m² [27, 32].

*Benefits in Diabetic Nephropathy*

It has been shown that GLP-1Rs are expressed in various cell types within the kidney, including proximal tubular cells, glomeruli, and vascular smooth muscle cells. In preclinical studies, GLP-1R has been localized at distinct sites throughout the kidney [26, 33].

Studies conducted in animal models have reported the presence of GLP-1R messenger RNA (mRNA) in the glomerulus and the initial portion of the proximal convoluted tubules, with no detectable expression in other regions of the nephron.

While the mechanisms underlying the potential renoprotective effects of GLP-1RAs remain unclear, research has hinted at a possible role for metabolites of endogenous GLP-1, such as GLP-1(9-37) and GLP-1(28-37). In animal studies, these metabolites failed to influence glucose metabolism but were linked to reduced expression of renal tubular injury markers and decreased tubulointerstitial damage, as evidenced by diminished accumulation of macrophages and T cells in the kidneys [33, 34].

Additionally, it appears that GLP-1RAs may have both direct and indirect effects on kidney function. The direct impact involves a more specific effect on the kidneys themselves.

The indirect mechanisms of the beneficial effects of glucagon-like peptide-1 receptor agonists (GLP-1RAs) remain uncertain, but it is possible that they are multifaceted, involving a combination of factors such as weight loss, blood pressure reduction, and glycemic control [31].

Research has shown that GLP-1RAs modestly reduce levels of low-density lipoprotein (LDL) cholesterol, total cholesterol, and triglycerides, but do not significantly impact high-density lipoprotein (HDL) cholesterol levels [27].

The acute administration of GLP-1R agonists is likely to increase effective renal plasma flow and glomerular filtration rate (GFR) in healthy individuals, primarily due to a temporary increase in blood pressure, which is mediated by an increase in heart rate and cardiac output. Conversely, chronic administration of GLP-1R agonists tends to lower blood pressure, promote natriuresis, and influence renal risk factors to support the maintenance of renal function [27, 31].

Several direct proposed mechanisms by which GLP-1RAs may exert renoprotective effects include:

Renal Tubular Effects: GLP-1 is thought to act on the renal tubule by promoting natriuresis and diuresis through inhibition of sodium-hydrogen exchanger 3 (NHE3) at the brush border of the proximal tubule. Studies in healthy subjects and those with type 2 diabetes have shown that infusion of GLP-1RAs such as lixisenatide, exenatide, and liraglutide reduces urinary sodium reabsorption and increases proximal urinary sodium excretion [26, 33].

Renal Haemodynamic Effects: The impact of GLP-1 on glomerular haemodynamics is unclear, with some studies suggesting that it may improve sodium and water handling and increase glomerular filtration rate (GFR) in rats, while others have found no effect on renal haemodynamics in humans [27]. 

Reduction in Renal Oxidative Stress: GLP-1 is also thought to have antioxidant properties, which may help to reduce the progression of diabetic kidney disease (DKD). For example, the administration of liraglutide to GLP-1 receptor knockout mice delayed the progression of DKD by reducing mesangial expansion and glomerular superoxide levels [26, 33].

Cyclic Adenosine Monophosphate-Protein Kinase A (cAMP-PKA) Pathway Activation: Activation of the cAMP-PKA pathway has been shown to reduce reactive oxygen species (ROS) production in the diabetic kidney, and GLP-1R activation leads to stimulation of this pathway, which has antioxidant effects [27].

Renin-Angiotensin-Aldosterone System (RAAS) Modulation: Activation of the intrarenal RAAS is a known pathogenic mediator of DKD, and there is evidence that GLP-1R agonists can reduce markers of renal RAAS activation, including angiotensin II levels and its deleterious effects in the glomerulus [27].

Additionally, studies in high-fat diet rats (HFD) have shown that liraglutide restored kidney mitochondrial function by increasing the biogenesis of genes encoding proteins involved in mitochondrial respiration and increasing uncoupling protein 2 levels [27, 33].

Recent clinical trials on kidney-protective effects of GLP-1 receptor agonist

Previous cardiovascular outcomes trials (CVOTs) conducted with GLP-1RAs such as liraglutide, subcutaneous semaglutide, dulaglutide, and efpeglenatide included renal outcomes as prespecified secondary endpoints in their studies [35, 36].

Despite the lack of a dedicated kidney outcomes trial specifically investigating the reno-protective effects of glucagon-like peptide-1 receptor agonists (GLP-1RAs), there is an ongoing clinical trial, FLOW, that aims to evaluate the kidney-protective effects of semaglutide in individuals with chronic kidney disease (CKD) and type 2 diabetes (T2D). Additionally, FLOW will assess the impact of semaglutide on cardiovascular mortality [32, 33].

The ELIXA trial found that lixisenatide reduces cardiovascular events in patients with type 2 diabetes and acute coronary syndrome as compared to placebo. A subsequent analysis of the trial data showed that lixisenatide reduced albuminuria in patients with macroalbuminuria by 39.18% over a median follow-up period of 108 weeks. Additionally, lixisenatide reduced the risk of new-onset macroalbuminuria by 20% compared to placebo but did not affect the risk of adverse renal events or doubling of serum creatinine [35, 36].

The LEADER trial found that liraglutide reduced the risk of kidney disease progression in patients with type 2 diabetes and cardiovascular disease. The study showed that liraglutide reduced the risk of new-onset persistent macroalbuminuria, a marker of kidney damage, by 26%. Additionally, liraglutide reduced the risk of kidney-related events such as the doubling of serum creatinine and the need for renal replacement therapy. Another study, LIRA-RENAL, found that liraglutide did not significantly affect kidney function in patients with moderate renal impairment, but did reduce urinary albumin-to-creatinine ratio by 17%. Overall, these studies suggest that liraglutide may have potential benefits for kidney health in patients with type 2 diabetes [35, 36].

The SUSTAIN-6 trial was a clinical study that tested the safety and efficacy of semaglutide. The study found that semaglutide was non-inferior to a placebo in terms of cardiovascular safety, and it also showed a significant reduction in the risk of nephropathy (kidney damage) [26, 35]. Specifically, semaglutide reduced the risk of persistent macroalbuminuria (seen in 2.5% compared to 4.9% in the placebo group) [36, 37].

The EXSCEL trial tested the cardiovascular safety of exenatide in 14,752 patients with type 2 diabetes. The results showed no significant difference in kidney function (eGFR) or new kidney damage (macroalbuminuria) between exenatide and placebo; a 15% lower risk of severe kidney damage with exenatide compared to placebo [35, 36].

The Harmony Outcomes trial, a large-scale study, tested the cardiovascular safety of albiglutide in patients with type 2 diabetes and cardiovascular disease. The results showed that albiglutide did not significantly differ from a placebo in terms of eGFR decline or renal events [35, 36]. However, when compared to insulin glargine, the GLP-1 receptor agonist dulaglutide demonstrated a more favorable effect on kidney function in patients with type 2 diabetes and moderate-to-severe kidney disease [38].

In the REWIND trial, dulaglutide was found to reduce the risk of kidney damage by 15% compared to a placebo in patients with type 2 diabetes and a history of cardiovascular disease or high-risk factors. This benefit was primarily driven by a significant reduction in the development of new kidney damage, as measured by albuminuria [37, 39].

These trials suggest that GLP-1 receptor agonists may have beneficial effects on kidney function and reduce the risk of kidney damage in patients with type 2 diabetes and cardiovascular disease or kidney disease.

Interestingly, studies have shown that human GLP-1 analogues such as liraglutide and semaglutide have a more favorable risk-benefit profile for major adverse cardiovascular events (MACE) and renal outcomes compared to exendin-4-based drugs like exenatide and lixisenatide. This may be due to their unique properties: they are resistant to renal elimination due to their large molecular weight or noncovalent binding to albumin, whereas exendin-4-based drugs are eliminated by the kidneys. Additionally, exendin-4 analogues are susceptible to degradation by dipeptidyl peptidase-4 (DPP-4), which could explain why GLP-1 analogues have been shown to have greater cardiovascular and renal protective effects [27].

The FDA warns that liraglutide may increase the risk of kidney damage, particularly in patients with heart failure. However, a recent analysis found no evidence of worsening kidney function in patients with heart failure who took liraglutide [30, 40].

Post-marketing reports have linked GLP-1 receptor agonists, including liraglutide, to acute kidney failure and worsening chronic kidney disease, sometimes requiring dialysis. These cases often occurred with gastrointestinal side effects like nausea, vomiting, and diarrhea, leading to dehydration. Another possible explanation is that GLP-1 receptor agonists alter blood flow to the kidneys [30, 40].

Rare cases of acute kidney injury (AKI) and interstitial nephritis have been reported after taking GLP-1 receptor agonists. However, subsequent studies have not confirmed these findings, and animal studies have shown that GLP-1 receptor agonists may actually protect against AKI [40].

The kidney function of patients taking these medications should be monitored closely and their doses adjusted carefully if necessary.

Neuroprotective benefits 

GLP-1 agonists have certain outcomes on memory, learning, and neuroprotection. The hippocampus is a part of the brain involved in spatial learning and memory and the expression of GLP-1 receptors has been found in the hippocampus of rats and mice, thereby improving different aspects of learning and memory [41]. 

Effects in Alzheimer’s disease 

Alzheimer's disease (AD) is characterized by the neurodegeneration of cholinergic neurons in the hippocampus. Research in rats has shown that administering GLP-1 receptor (GLP-1R) agonists to the hippocampus can prevent spatial learning and memory impairments caused by amyloid β (Aβ), a protein implicated in AD [42]. There is a pathophysiological link between type 2 diabetes mellitus (T2DM) and AD, with T2DM identified as a risk factor for AD [43]. Insulin signaling, impaired in T2DM, is also desensitized in AD brains. Insulin not only regulates blood glucose but also promotes neuronal growth, repair, and cognitive functions. Similarly, GLP-1 in the brain acts as a growth factor, enhancing cell growth, repair, and neuroprotection [44].

Studies have shown that GLP-1R agonists, such as exendin-4, liraglutide, and lixisenatide, can rescue spatial memory impairments induced by Aβ in rats [45]. Exendin-4, a potential GLP-1R ligand, protects neurons from damage through cAMP-mediated pathways [46]. Overexpression of GLP-1R in the hippocampus improves neurite growth and learning, while GLP-1R knockout results in cognitive deficits [47]. Exendin-4 also prevents Aβ-induced apoptosis by maintaining Bcl-2 levels and inhibiting caspase-3 activation, crucial factors in AD pathogenesis [48]. The cAMP/PKA signaling pathway plays a key role in exendin-4's protective effects against Aβ-induced memory impairments, highlighting the therapeutic potential of GLP-1R agonists in AD treatment [49].

Effects in Huntington’s Disease 

Huntington’s disease (HD) is characterized by severe neurodegeneration resulting from a mutation in the huntingtin protein (HTT). This mutation leads to increased oxidative stress and improper autophagic clearance of HTT, causing neurotoxicity and degeneration [50]. The prevalence of type 2 diabetes mellitus (T2DM) is higher in HD patients, suggesting that impaired insulin sensitivity may contribute to neurodegeneration in HD. Overexpression of mutant HTT disrupts insulin signaling and promotes neuronal apoptosis in human neuronal cells. Treatment with liraglutide improves insulin sensitivity and cell viability by reducing neuronal glucotoxicity, oxidative stress, and mutant HTT levels [51].

Although research on the effects of GLP-1 in HD is limited, a study using a mouse model demonstrated that peripherally administered exendin-4 improved motor coordination and general activity levels in HD mice, extending their survival compared to controls [52]. Exendin-4 also slowed the decline in motor performance typically seen in HD progression. Metabolic improvements, such as better glycemic control and enhanced insulin-stimulated glucose uptake, were observed alongside improved cell architecture. These benefits were associated with a reduced number of mutant HTT aggregates in islet and cerebrocortical cells [53].

Effects in Parkinson’s Disease

GLP-1 analogs have demonstrated success in treating Parkinson’s Disease (PD), which is characterized by the degeneration of dopaminergic neurons. This condition can be replicated in experimental animals using the dopaminergic neurotoxin MPTP (1-Methyl-4-phenyl-1,2,3,6-tetrahydropyridine) [54]. Infusing exendin-4 into the lateral ventricle of mice provided protection against MPTP-induced damage to the dopaminergic system and prevented locomotor deficits associated with dopamine deficiency. Exendin-4 was also shown to increase survival and levels of tyrosine hydroxylase, the key enzyme in dopamine production [55].

Other GLP-1 receptor agonists, such as liraglutide and lixisenatide, have exhibited superior neuroprotective effects compared to exendin-4 in the MPTP mouse model of PD. Studies have shown that liraglutide and semaglutide enhance locomotor and exploratory activities impaired by MPTP, improving bradykinesia, movement coordination, and balance in mice [56, 57].

Effects in stroke

Individuals with diabetes have a 2.9 times higher risk of stroke, particularly ischemic stroke, compared to the general population, and their risk of death after a stroke is significantly elevated. The pathophysiology of stroke involves the apoptosis of cortical and striatal neurons [58].

Research has shown that intracerebral administration of exendin-4 provides neuroprotection and improves locomotor activity after a stroke in rats, with these effects confirmed in GLP-1R knockout mice, indicating the involvement of GLP-1R [54]. Additional studies demonstrated that transvenous administration of exendin-4 offers neuroprotection against ischemic injury in mice following middle cerebral artery occlusion (MCAO). The neuroprotective efficacy of exendin-4 was also observed in both young healthy and aged diabetic/obese mice. Moreover, administering exendin-4 before MCAO was found to result in neuroprotection [59].

Effects in peripheral neuropathy

About 60-70% of people with diabetes have some degree of neurological damage, specifically neuropathies that lead to impaired sensation in the hands and/or feet, reduced gastric motility, or carpal tunnel syndrome.

Therapy for the neurological damage caused by prolonged hyperglycemia and the associated metabolic disturbances is symptomatic relief [60].

GLP-1 expression has been observed in neurons in the nodose ganglion, indicating a role for GLP-1 in peripheral neurotransmission. This was observed in studies where GLP-1 analog administration into the portal vein activated the enteric nervous system and triggered cerebral control of peripheral functions such as glucose utilization. The nodose ganglion includes sensory afferents important to various autonomic reflexes, and these nerves may be damaged by different factors, including diabetes mellitus, toxic compounds, and drugs [61].

Ingestion of large amounts of pyridoxine (vitamin B6) causes peripheral sensory neuropathy in humans, a condition that mimics diabetic peripheral neuropathy by damaging large sensory neurons [60].

In pyridoxine-induced peripheral neuropathy in non-diabetic rodents, GLP-1 and subcutaneous exendin-4 were observed to partially protect against several pyridoxine-induced functional and morphological damages and to promote normalization of axonal size [60].

More studies are needed to determine what functions GLP-1 plays in peripheral neuropathies. 

Gastrointestinal effects 

Major setbacks of earlier antidiabetic agents include hypoglycemia and weight gain. Glucagon-like peptide-1 agonists (GLP-1As) have opened up new possibilities for controlling hyperglycemia in patients with Type 2 Diabetes mellitus [62]. An oral glucose load provokes a higher insulin response compared with an equivalent dose of glucose given intravenously. Oral glucose causes a release of incretins which are gut hormones that amplify the glucose-induced insulin secretion. This is called the incretin effect. The GLP-1 effect is dependent on the levels of glucose such that this effect is accentuated when glucose levels are high and attenuated when glucose levels decline. The GLP-1 receptor (GLP-1R) is expressed in the pancreatic islet α and β cells and in peripheral tissues, including the nervous system, heart, kidney, lung, and gastrointestinal tract [63]. This accounts for its widespread physiological organ-system effects in ultimately lowering blood glucose levels.

Impacts on Gastric Emptying and Satiety

GLP-1 agonists, including exenatide, liraglutide, lixisenatide, albiglutide, and taspoglutide can stimulate insulin secretion (similar to other insulin secretagogues), improve peripheral insulin resistance, decrease body weight, and reduce the risk of hypoglycemia for Type 2 diabetic patients [63]. It also delays gastric emptying by affecting gut motor function and by antral distension through specialized nervous system pathways [64]. Other well-described central nervous system (CNS) actions include reduction of gastric acid secretion, increased satiety, reduction in food craving, and lower preference for energy-dense foods [65, 66]. This gut-brain (neuropeptidergic) mechanism maintains the ileal brake mechanism which is the inhibitory feedback system to control the transit of a meal through the gastrointestinal tract so as to efficiently digest and absorb nutrients [67]. Hence, Type 2 diabetes patients on GLP-1 therapy are less hungry due to a decrease in gastric emptying and inhibition of feeding by a CNS mechanism.

Beyond glucose maintenance, there is accumulating evidence to support the beneficial role of GLP-1 agonists in the gastrointestinal tract. Hunt JE et al reviewed data investigating GLP-1 as a novel treatment for intestinal diseases, such as inflammatory bowel diseases, short-bowel syndrome, intestinal mucositis, and coeliac disease [65]. These diseases represent pathological processes like immune cell infiltration, epithelial cell damage, and chronic inflammation. Possible beneficial mechanisms for these diseases can be attributed to GLP-1's influence on gastric emptying, together with its anti-inflammatory properties and its intestinotrophic effect [65]. Furthermore, rats treated with exendin-4, a GLP-1 agonist, were found to have increased intestinal absorption and gut permeability mediated by increasing the mucosa surface area; and also reported increased intestinal weights following GLP-1 analogue treatment [68]. GLP-1 mimetics also play a role in regulating the immune response in the gastrointestinal system by reducing the production of pro-inflammatory cytokines [69], GLP-1 mimetics have been found to enhance Brunner gland function and therefore barrier-protection of the gut [64].

Gastrointestinal side effects are the commonest reported treatment-related effects of GLP-1 agonists in many clinical trials [62]. They include nausea, vomiting, diarrhea, and less commonly constipation [64, 67]. These side effects are induced by various mechanisms majorly activation of central and peripheral GLP-1 receptors [67].

Research has found that all GLP-1 RA regimens significantly increased the incidence of side effects on the gastrointestinal system, compared with placebo or other anti-diabetic treatments commonly used [64, 70]. Furthermore, recent clinical evidence suggests that the occurrence of gastrointestinal adverse events was different with diverse dose regimens, treatment adherence, and combination with other anti-diabetic agents like metformin or insulin [70]. However, these adverse effects have also been noted to decline with time.

In one study, patients treated with exenatide had the highest incidence of nausea and vomiting as opposed to other treatments, while patients with liraglutide had the highest incidence of diarrhea indicating that exenatide and liraglutide had the more treatment-related gastrointestinal adverse events definitely than other treatments [65]. In a more recent study, taspoglutide had the maximum probability of causing vomiting and nausea, whereas lixisenatide had the maximum probability of causing the development of diarrhea versus other treatments [62]. Other side effects such as abdominal discomfort, eructation, flatulence, and dyspepsia have also been recorded to occur with GLP-1 treatment, although these have been found to be less common [67]. 

Safety and tolerability

Since GLP-1 receptor agonists mimic the action of a peptide produced in the gastrointestinal (GI) tract, the most reported side effects are GI, including nausea which is the most common occurring in 25-60% of patients in clinical trials, vomiting in 5-15% of patients, and diarrhea in 10-20% of patients [71].

Long-acting GLP-1 receptor agonists (GLP-1RAs) like liraglutide tend to have more pronounced and persistent gastrointestinal effects compared to short-acting ones like exenatide. This is primarily due to their prolonged action and continuous activation of GLP-1 receptors [72].

Injection site reactions and erythema have been observed to occur more frequently with once-weekly administration of exenatide compared to its other form which requires twice-daily dosage. Similarly, injection site reactions are more prevalent with once-weekly exenatide or dulaglutide than with once-daily liraglutide. This difference is likely due to the extended-release mechanisms and larger injection volumes used in once-weekly formulations [73].

GLP-1 receptor agonists have also been shown to not elevate the risk of hypoglycemia when used independently, although their combination with insulin or sulfonylureas may increase this risk. In these cases, it's often recommended to reduce the dose of insulin or sulfonylurea to mitigate the risk of hypoglycemic episodes [73, 74].

Despite initial concerns regarding impacts on pancreatic and thyroid tissues, several meta-analyses and large cohort studies have not established a causal link between GLP-1 receptor agonists and pancreatitis, pancreatic cancer, or thyroid cancer over years of follow-up [75, 76].

Concerns have been raised about the potential for GLP-1 agonists to delay gastric emptying. This raises the risk of regurgitation and aspiration. Thus, as a precaution for elective procedures, it is suggested that GLP-1 agonists be discontinued temporarily. This recommendation applies regardless of the indication for which the medication is prescribed.

It is interesting to note that a meta-analysis conducted on all 15 GLP-1RA drugs regarding significant efficacy in reducing HbA1c (glycated hemoglobin) levels compared with placebo in adults with Type 2 diabetes revealed Tirzepatide as the drug which caused the most significant reduction [8].

Regarding long-term tolerability, the rate of discontinuation of GLP1 agonists due to adverse events is generally low (around 10%) and comparable to or slightly higher than other diabetes medications [75, 77]. Since tolerability varies between individuals and specific GLP1 receptor agonists, it is important to ensure proper patient education about potential side effects and their typically transient nature to improve tolerability and adherence [78, 79].

## Conclusions

GLP-1 agonists have proven highly effective in managing type 2 diabetes and related metabolic disorders, offering benefits such as improved glycemic control, weight loss, enhanced cardiovascular and renal health, and potential neuroprotective effects. Emerging evidence suggests their utility in treating neurodegenerative diseases and reducing stroke risk. Further research, especially involving human subjects, is essential to fully understand their long-term effects and broader applications. 
